# Rolling of soft microbots with tunable traction

**DOI:** 10.1126/sciadv.adg0919

**Published:** 2023-04-21

**Authors:** Yan Gao, Brennan Sprinkle, Ela Springer, David W. M. Marr, Ning Wu

**Affiliations:** ^1^ Materials Science and Engineering Program, Colorado School of Mines, Golden, CO, USA.; ^2^ Department of Applied Mathematics and Statistics, Colorado School of Mines, Golden, CO, USA.; ^3^ Department of Chemical and Biological Engineering, Colorado School of Mines, Golden, CO, USA.

## Abstract

Microbot (μbot)–based targeted drug delivery has attracted increasing attention due to its potential for avoiding side effects associated with systemic delivery. To date, most μbots are rigid. When rolling on surfaces, they exhibit substantial slip due to the liquid lubrication layer. Here, we introduce magnetically controlled soft rollers based on Pickering emulsions that, because of their intrinsic deformability, fundamentally change the nature of the lubrication layer and roll like deflated tires. With a large contact area between μbot and wall, soft μbots exhibit tractions higher than their rigid counterparts, results that we support with both theory and simulation. Upon changing the external field, surface particles can be reconfigured, strongly influencing both the translation speed and traction. These μbots can also be destabilized upon pH changes and used to deliver their contents to a desired location, overcoming the limitations of low translation efficiency and drug loading capacity associated with rigid structures.

## Teaser

Soft microbots based on magnetic Pickering emulsions exhibit tractions higher than their rigid counterparts.

## INTRODUCTION

External control of microscale robots has attracted notable interest because of the desire to direct in vivo devices (*1*, *2*). However, compared with microbot (μbot) manipulation methods, such as chemical (*3*, *4*), electric (*5*, *6*), light (*7*, *8*), or acoustic techniques (*9*), magnetic actuation methods have several advantages, including noninvasiveness, fast response times, high directionality (*10*, *11*, *12*), and excellent biocompatibility (*13*). As a result, magnetic field–assisted drug delivery efforts can be traced back to the ‘80s (*13*, *14*), with now multiple magnetic μbot approaches developed (*15*), including helical swimmers (*16*), microwalkers (*17*), undulating chains (*18*), flapping nanomagnets (*19*), reconfigurable swarms (*20*), and surface rollers (*21*).

Unlike microscopic swimmers, rolling μbots use friction with available surfaces to move. Hard μbots (*22*) in liquid media, however, roll differently than in dry environments because a liquid lubrication layer between the μbot and wall leads to substantial slip. Lubrication theory predicts that a rigid rolling microsphere must slip near a solid substrate with a maximum rolling velocity of only one-fourth the speed with no slip present (*23*). Overcoming this theoretical limit requires new approaches if efficient microscale locomotion is to be achieved, an issue particularly important for microenvironments where the surface-to-volume ratios are high. Inspired by tank treading, where a large and flat contact area faces the translation surface without slip, we introduce soft μbots based on Pickering emulsions stabilized by magnetic particles. These fully and partially covered emulsion droplets deform near walls, roll like deflated tires, and exhibit larger traction and translate faster than their rigid counterparts. In addition, the particle distribution at the emulsion droplet surface can be reconfigured using different combinations of alternating current (AC) and direct current (DC) magnetic fields to enable direct control of droplet traction. Last, pH-triggered destabilization of the μbots is demonstrated as a potential approach to targeted delivery. Because of their softness and the liquid core, our soft μbots have the potential for increased biocompatibility, high drug loading capacity, and enhanced transport through physical constraints that may exist in vivo (*24*, *25*).

## RESULTS

### High traction of deformable droplets

**Fig. 1.**
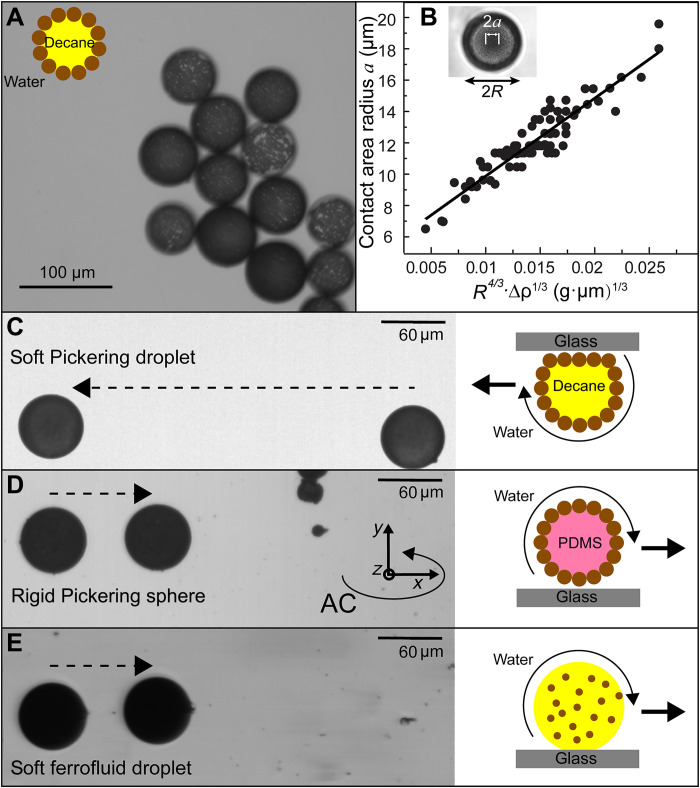
The rolling of deformable soft droplets. (**A**) Pickering decane-in-water emulsion droplets are partially or fully covered with modified 1-μm polystyrene-Fe_3_O_4_ composite beads. (**B**) The contact area radius *a* of the droplets depends on their radius *R*. Fit is based on Eq. 1. Inset: The image of a deformed droplet. Rolling of a (**C**) soft Pickering droplet, (**D**) a rigid Pickering sphere, and (**E**) a soft ferrofluid droplet over 8.5 s under a rotating magnetic field (*H*_0_ = 2.5 mT and *f* = 20 Hz) along the *x-z* plane.

To make them amphiphilic, we first coat the surfaces of carboxyl-functionalized superparamagnetic microparticles with decylamine. As shown in fig. S1, successful attachment is confirmed with peaks in the Fourier transform infrared spectra appearing at 2850 cm^−1^ (C─H stretch) and 725 cm^−1^ (C─H rock bond). The zeta potentials are also changed from −60 ± 5 to −35 ± 4 mV upon surface modification. We then produce stable Pickering oil-in-water (o/w) emulsions by homogenizing the particle, decane, and water mixture. Depending on the homogenization speed and initial particle concentration, the surface coverage of particles can be tuned from 30 to 100% with a broad size distribution (Fig. 1A). The fabricated Pickering emulsions remain stable for at least several months, allowing us to investigate their actuation under magnetic fields conveniently.

Since decane is lighter than water, droplets float to the top slide and deform because of their softness. Under high magnification, the circular contact area can be clearly seen (Fig. 1B, inset) with all particles in focus within the contact plane (fig. S2). The contact area radius is governed by the Hertzian model (*26*), where the droplet deforms because of the competition between buoyancy and elastic forces

$$a={\left(\frac{3WR}{4E}\right)}^{\frac{1}{3}}={\left(\frac{\pi g\text{Δ}\rho }{E}\right)}^{\frac{1}{3}}R^{\frac{4}{3}}$$ (1)

with *W* as the buoyancy force, *g* as the gravitational constant, and ∆ρ as the density difference between water and droplets consisting of decane and surface magnetic particles. *E* is the effective stiffness between the elastic properties of two materials

$$\frac{1}{E}=\frac{1-v_{1}^{2}}{E_{1}}+\frac{1-v_{2}^{2}}{E_{2}}$$ (2)

where *v*_1_ and *v*_2_ are the Poisson’s ratios and *E*_1_ and *E*_2_ are Young’s moduli of the droplet and glass slide, respectively. In our experiments (Fig. 1B), we find that the contact area radius *a* is independent of the particle surface coverage and scales as *a* ∝ *R*^4/3^, as expected from Eq. 1. The impact of droplet radius and surface particles on the droplet density (*27*) has been included in the original Hertzian model calculations in Fig. 1B. On the basis of Eq. 2, we calculate the effective Young’s modulus of droplets *E*_1_ ~ 139 Pa, consistent with literature values for Pickering emulsions (*28*). This agreement also indicates that the adhesion force between the droplet and glass slide is negligible because *a* would otherwise scale with *R*^2/3^ (*29*). Moreover, when we tilt the glass slide ~4°, droplets roll off, confirming that the adhesive force is minimal.

The softness and deformability of Pickering droplets notably affect their rolling behavior. Here, we apply a two-dimensional (2D) rotating magnetic field (fig. S3) along the *x-z* plane, $H=H_{0}(\cos\omega_{M}t\hat{x}+\sin\omega_{M}t\hat{z})$, where *H*_0_ is the field strength and *f* = ω*_M_*/2π = 20 Hz is the field frequency. Note that the step-out frequency of the soft droplets is 1 to 3 Hz, which decreases with increasing droplet radius. As with our previous work on μbots assembled from hard beads (*30*), soft droplets roll because of the broken symmetry in hydrodynamics near the top glass slide (Fig. 1C and movie S1). Movie S2 also shows that the contact area of the static and rolling droplets remains constant. Here, the droplet surface particles produce a tank-treading motion and roll like a deflated tire. For comparison, we study analogous rigid μbots by replacing the liquid core with cross-linked poly(dimethylsiloxane) (PDMS) while retaining the outer particle shell, expecting an elastic modulus ~26,000 times greater than the soft ones (*31*). Although these rigid spheres are heavier than the water medium and settle to the bottom glass substrate, no deformation can be observed. Under identical fields, these rigid Pickering spheres roll like fully inflated tires; however, they roll ~3× more slowly than soft droplets (Fig. 1D and movie S1), although they are of similar size and have the same magnetic particle surface coverage. Note that the translation direction of the rigid sphere is opposite the Pickering droplet because one is adjacent to the bottom substrate, while the other floats to the top.

For further comparison, we have fabricated soft droplets with a ferrofluid liquid core composed of 5 wt % 10-nm Fe_3_O_4_ nanoparticles. These roll at a similar speed as rigid Pickering spheres, ~3× slower than the soft Pickering droplets. Soft ferrofluid droplets roll slowly because the magnetic nanoparticles are suspended in the droplet and are free to rotate in the liquid core under applied fields. As a result, the net torque transmitted to the droplet is small because of viscous dissipation. Therefore, although the magnetic particle loading in the ferrofluid droplet is much higher than in the Pickering droplet, it is not a very efficient μbot. The difference in speed between rigid and soft spheres in Fig. 1 (C and D), however, poses interesting questions regarding the role of deformation in μbot translation.

**Fig. 2.**
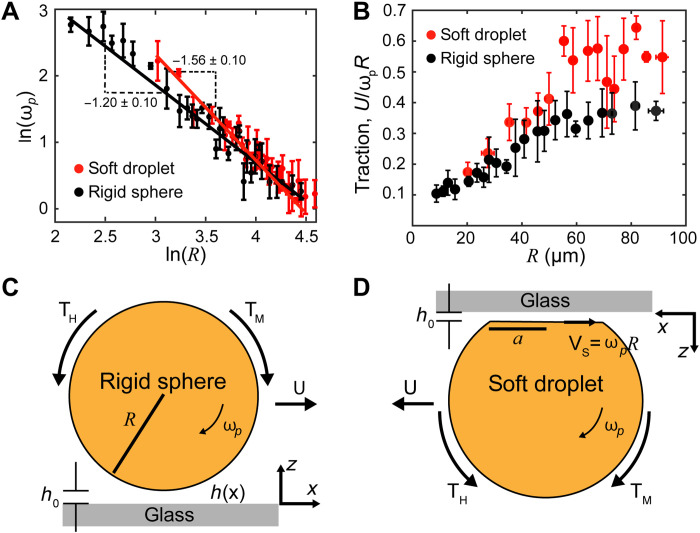
The velocity and traction of soft and rigid μbots. (**A**) Angular velocities of rigid and soft Pickering droplets under identical magnetic fields (*H*_0_ = 2.5 mT and *f* = 20 Hz) with the slopes of the fitted lines indicated. (**B**) Tractions of rigid and soft Pickering droplets. The error bars in (A) and (B) around each data point are determined from five different measurements. Schematic of a (**C**) rigid sphere and (**D**) soft droplet rolling under an AC magnetic field. **T**_**H**_ and **T**_**M**_ are the hydrodynamic and magnetic torques. Surface particles in both cases are not shown for simplicity.

To fully characterize the rolling of soft and rigid Pickering droplets, we measure the translational *U* and angular rotation ω_p_ velocities for different droplet sizes with 100% surface coverage. Minor but visible defects in surface particle packing allow for accurate tracking of rotation rates. The dependence of the droplet velocity on radius and actuation frequency is shown in fig. S4. As shown in Fig. 2 (A and B), the angular velocities of soft and rigid droplets show different size dependencies, with the traction of soft droplets higher than that of solid ones. Here, we define the traction γ* = *U*/*ω_p_*R* as a means of characterizing rolling; a value of γ = 1 corresponds to no slip, while μbots with γ = 0 would not translate.

The rolling behavior of rigid droplets can be understood by classical hydrodynamic lubrication theory (*23*). In this, the hydrodynamic torque *T_H_* consists of torques due to translation and rotation

$$T_{H}=T_{H}^{t}+T_{H}^{r}=8\pi R^{3}\eta \omega (T_{H}^{r*}+\gamma T_{H}^{t*})$$ (3)

where η is the solvent viscosity. When the separation between the sphere and the wall is small (*h*_0_/*R* ≪ 1), the correction factors $T_{H}^{t^{*}}$ and $T_{H}^{r^{*}}$ can be expressed as

$$T_{H}^{r*}=\frac{2}{5}\ln\left(\frac{h_{0}}{R}\right)-0.3817\;\mathrm{and}\;T_{H}^{t*}=-\frac{1}{10}\ln\left(\frac{h_{0}}{R}\right)-0.1895$$ (4)

The magnetic torque **T**_**M**_ is the sum of torques generated on all surface particles

$$T_{M}=\langle \mu_{s}m\times H\rangle N_{p}\mu_{s}\chi^{\prime \prime }V_{0}H_{0}^{2}=4R^{2}\mu_{s}\chi^{\prime \prime }V_{0}H_{0}^{2}/r_{p}^{2}\hat{y}$$ (5)

where ⟨·⟩ corresponds to the average over one period of the rotating field. **m** is the magnetic dipole moment of one particle, **H** is the applied field, and μ_s_ is the solvent permeability. *N*_p_ = 4$\pi R^{2}/{\pi r}_{p}^{2}$ is the total number of particles on the droplet surface, where *r*_p_ is the radius of the magnetic particle. The imaginary part of the magnetic susceptibility χ″ characterizes the phase lag between the induced dipole and the field that generates the torque. *V*_0_ is the volume of one particle, and *H*_0_ is the field strength.

At steady state, the balance between the hydrodynamic and magnetic torques yields a scaling between the angular velocity and the droplet size ω_p_ ∝ *R*^−1^[* −*ln (*h*_0_/*R*)]^−1^ ≈ *R*^−1.36^ (section S1 and fig. S5) close to the scaling ω_p_ ∝ *R*^−1.2^ observed in experimental measurements (Fig. 2A). Minor discrepancies can be explained by the neglect of the pressure force within the lubrication layer, which effectively makes the gap *h*_0_ larger than expected from a balance of gravitational and electrostatic forces only.

Figure 2B shows that rigid droplets have lower traction than soft ones. In this, the entrained thin liquid film between the wall and rigid sphere acts as an effective lubrication layer, making the rolling inefficient. However, the situation is different for soft droplets, which deform and maintain a substantial Hertzian contact area with the wall (Fig. 2D). Unlike the wedge-shaped liquid film *h*(*x*) between the rigid droplet and the wall, the confined liquid for soft droplets has an almost constant thickness *h*_0_ extending across the entire contact area, making the lubrication effect only dominant in the regions near the contact edges (*32*). Moreover, because *a* ≫ *h*_0_, we can safely assume that the net flux of fluid within the gap, $q=\int_{0}^{h_{0}}v_{x}dz$, where *v_x_* is the fluid velocity along the *x* axis within the gap, is very small. Therefore, any volume of fluid displaced by the droplet is assumed to flow around the droplet away from the wall rather than through the gap. After solving the velocity profile within the gap and equating the shear and pressure forces for the soft droplet, we can conclude (section S2) that the difference between the droplet translation velocity *U* and the tank treading velocity of surface particles *V*_s_ = ω_p_*R* is small

$$U-V_{s}=U-\omega_{p}R=2q/h_{0}$$ (6)

This explains the large traction observed in soft droplets. An additional impact of μbot deformability is that the Hertzian contact can make the droplet very compliant with the substrate. As a result, the liquid flux within the gap may be extremely small, approaching the limit of dry friction with no slip. Therefore, the softness of the μbots can have a profound impact on the rolling of μbots.

### Numerical simulation

To further corroborate our theoretical analysis, we also performed numerical simulations where the droplet softness can be varied directly. Because of constraints in computational time, we choose droplets with a range of sizes between *R* = 4.61 and 19.78 μm, whose surfaces are densely covered by *N*_p_ 1-μm-diameter particles (e.g., *N*_p_ = 252 for *R* = 4.61 μm and *N*_p_ = 4002 for *R* = 19.78 μm). Here, *R* is reported without including particle size; therefore, the outermost radius of a droplet is *R*_o_ = *R* + 0.5 μm, assuming that particles are located with their midplanes at the liquid-liquid interface. While the droplet sizes that we consider are typically smaller than those used in our experiments, they are sufficient to investigate the effect of droplet softness on translation and traction because the ratios of the contact area to droplet radii are comparable to the larger droplets. Furthermore, numerical simulations allow us to decouple the softness of a droplet from its size and independently investigate its effects on droplet traction. To study the effect of droplet softness on locomotion, we vary the dimensionless contact radius

$$\bar{a}=\frac{a}{2r_{p}}={\left(\frac{3N_{p}m_{e}gR}{4\kappa_{\mathrm{bend}}}\right)}^{1/3}$$ (7)

where *m*_e_ is the effective mass of the particles and κ_bend_ is the effective bending modulus of the droplet, whose deformation is primarily controlled by the balance between κ_bend_ and gravitational energy. A softer droplet with a smaller κ_bend_ or a larger *R* has a larger dimensionless contact radius $\bar{a}$. In our simulations, we consider the following forces on the group of particles straddling the surface of a Pickering droplet of radius *R*: the magnetic force **F**^mag^ that drives the droplets, the hydrodynamic force **F**^hydro^, the interfacial forces **F**^tension^ and **F**^bend^ that constrain the particles to the interface and capture the droplet softness, and the gravitational force **F**^grav^ that balances **F**^bend^ to control deformation against the wall. The detailed expressions of those forces, our simulation method, and a table of parameters used to match experimental conditions can be found in Materials and Methods.

**Fig. 3.**
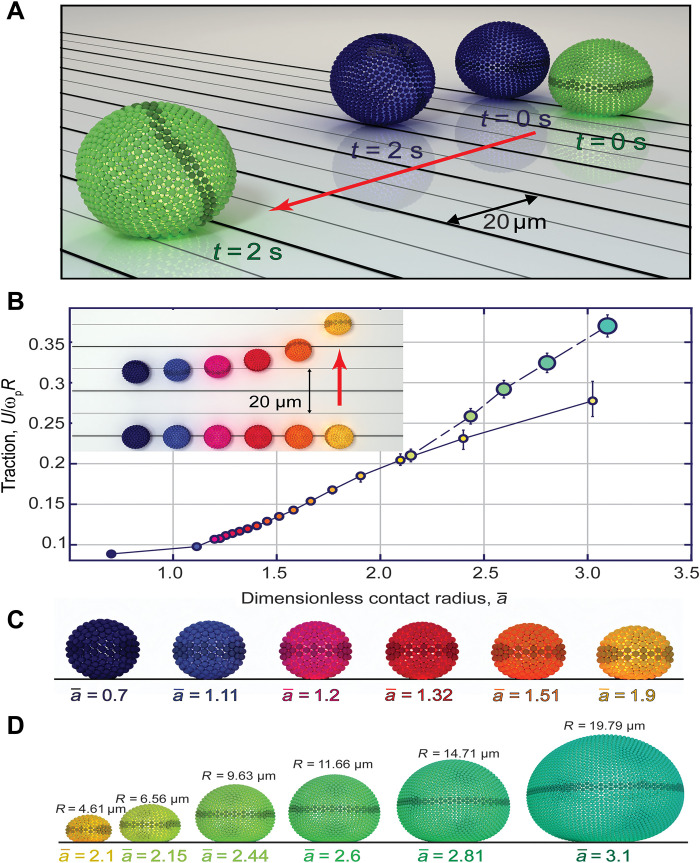
Numerical simulation on rolling of droplets with different softness and size. (**A**) Position of larger droplets with *R* = 9.6 μm at times *t* = 0 s and *t* = 2 s. The softer green droplet ($\bar{a}=2.43$) rolls 3.5× faster than the blue rigid droplet ($\bar{a}=1.09$; movie S3). Particles around the droplet equator are darkened to provide a visual indication of the droplet orientation. (**B**) Droplet traction γ versus dimensionless contact radius $\bar{a}$. The solid black curve shows the effect of varying $\bar{a}$ for a fixed droplet size *R* = 4.61 μm, where markers (also of fixed size) are colored from rigid droplets in indigo to soft droplets in yellow. The dashed black curve shows the effect of varying $\bar{a}$ by varying droplet size *R*, where marker sizes vary according to droplet size. Inset: The positions of six representative droplets (top) with varying $\bar{a}$ at times *t* = 0 s and *t* = 2 s. Grid lines on the substrate surface are spaced every 10 μm with the red arrow indicating the rolling direction. (**C**) Droplet side view equilibrated over 0.25 s with no applied magnetic forces. All droplets have the same radius *R* = 4.61 μm. (**D**) Droplets with varying *R* and contact radius $\bar{a}$ vary accordingly.

Each of our simulations begins with an initially spherical droplet formed by vertices on an icosphere (*33*) where particles are spaced approximately 2*r*_p_ apart and equilibrated with no applied magnetic force over 0.25 s to allow for elastic deformation due to gravitation. For convenience, we make the droplet heavier than the medium so it settles and rolls on the bottom substrate. Figure 3A and movie S3 show the rolling of rigid and softer droplets of the same size (*R* = 9.6 μm) in 2 s. Particles around the droplet equator are darkened to provide a visual indication of the droplet orientation. The softer green droplet ($\bar{a}=2.43$) rolls 3.5× faster than the blue rigid droplet ($\bar{a}=1.09$), leading to tractions of γ = 0.26 versus γ = 0.074. These values are consistent with our experimental values for small (*R* ≤ 10 μm) rigid droplets, although we cannot make soft droplets smaller than 10 μm with 1-μm magnetic particles.

Figure 3B shows results from our numerical studies on the simulated droplet traction γ = *U*/ω_p_*R *as a function of dimensionless contact radius $\bar{a}$. The error bars show the spread in traction values that we observe by using different parameter values representing the same value of $\bar{a}$, with details provided in Materials and Methods. The low variance in the error bars demonstrates that droplet traction is primarily controlled by the dimensionless softness $\bar{a}$. Here, we vary $\bar{a}$ both by fixing *R* = 4.61 μm and changing κ_bend_ (Fig. 3C) and by fixing κ_bend_ while changing *R* (Fig. 3D). Figure 3B shows that increasing $\bar{a}$ for a fixed droplet size (solid black curve) monotonically increases the droplet’s traction γ. The inset of Fig. 3B shows how far six representative droplets with fixed size but different κ_bend_ have traveled over a 2-s interval. Here, the softer droplets travel faster and have larger traction than more rigid ones. The softer droplet results in a larger traction of γ = 0.18, nearly 2× faster than the equal-sized rigid droplet with γ = 0.089 and consistent with the experimentally observed trend in traction values for increasingly soft droplets. However, beyond roughly $\bar{a}=2.1$, the rate of increase in γ begins to slow because of finite size effects from the fixed droplet size. By increasing $\bar{a}$ using the droplet radius instead (dashed black line), we avoid finite size effects at the cost of more expensive computations; however, we also see agreement between the two curves around $\bar{a}=2.1$, indicating that any method of varying $\bar{a}$ (droplet size or κ_bend_) produces the correct trend in γ so long as finite size limitations are avoided. Although the current simulation method prevents us from simulating even larger droplets within a reasonable amount of time, the black dashed curve in Fig. 3B shows that the traction increases with droplet size, consistent with experiments, and this trend depends only on the dimensionless Hertzian contact radius $\bar{a}$.

### Traction tuning by reconfiguring the distribution of surface particles

**Fig. 4.**
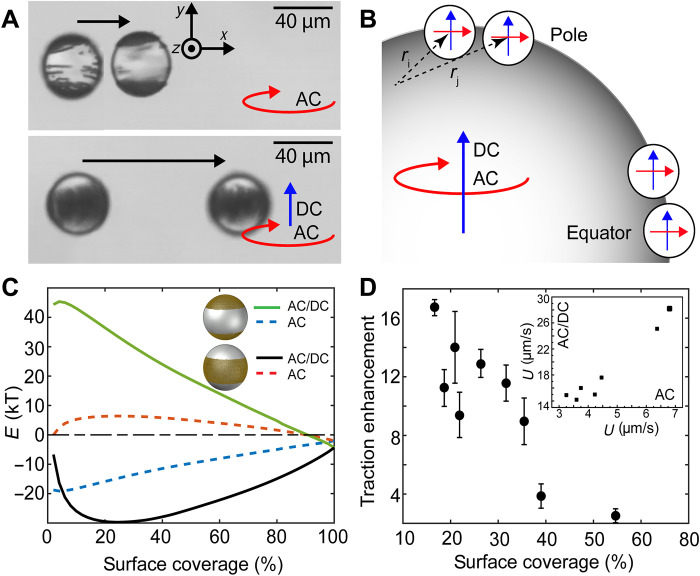
Reconfiguration of surface particles tunable by external fields. (**A**) Superimposed photos showing the reconfiguration of surface particles and the difference in velocities under different magnetic fields at a particle surface coverage of 40%. The AC field is 2.5 mT and 20 Hz, and the superimposed DC field is 2.5 mT. (**B**) Illustration of the induced dipoles between neighboring spheres located at the equator and the pole. Red arrows are dipoles induced by the rotating field, while blue arrows are dipoles induced by the DC field. (**C**) Calculated energies per particle for different magnetic fields and surface particle configurations. (**D**) Traction enhancement for the equatorial configuration. Inset: Measured velocities at 30% surface coverage. The error bars around each data point are SDs determined from five different measurements.

In addition to modifying the traction of soft μbots via their deformability, we can also tune it by reconfiguring the surface particle distribution on partially covered droplets. For example, when we apply a 2D 2.5-mT rotation field along the *x-z* plane, the initially uniformly distributed particles at 40% surface coverage migrate toward the two poles (Fig. 4A). When we superimpose a 2.5-mT DC field along the *y* axis, however, particles redistribute and accumulate at the equator. Here, the redistribution of surface particles is influenced by both the magnetic field and the confinement imposed by the curved interface. As shown in Fig. 4B, spheres located near the equator and the pole experience different dipolar interactions under the two different magnetic fields. When an *x-z* rotating field is applied, spheres near the equator experience a strong dipolar repulsion (Fig. 4B, red arrows) because the angle θ between the center connector **r***_ij_* = **r***_j_* − **r***_i_* and the field **H** is close to 90°. In comparison, spheres near the pole have a θ much smaller than the magic angle of 54.74° and experience a strong dipolar attraction. The situation changes, however, when a strong DC field is superimposed along the *y* direction. The DC field–induced dipolar interaction (Fig. 4B, blue arrows) is attractive for spheres near the equator because θ is close to zero, while it is repulsive for particle pairs near the pole. With a strong enough DC field, therefore, particles prefer to accumulate near the equator to maximize their alignment of dipoles with the DC field.

Quantitatively, we can also calculate the total magnetic energies of surface particles with different configurations. Under the combined DC (along the *y* axis) and AC rotating fields (along the *x-z* plane), $H=H_{xz}\cos(\omega_{M}t)\hat{x}+H_{y}\hat{y}+H_{xz}\sin(\omega_{M}t)\hat{z}$, the time-averaged pair interaction between spheres *i* and *j* can be expressed as (section S3)

$$\begin{array}{r} \langle E_{ij}(r)\rangle =\frac{m_{0}^{2}}{4\pi \mu_{s}r^{3}{\left[1-\frac{2\chi^{\prime }}{3}{\left(\frac{r_{p}}{r}\right)}^{3}\right]}^{2}}\cdot \{1-\frac{3[(r^{2}-r_{y}^{2})H_{xz}^{2}+2r_{y}^{2}H_{y}^{2}]}{2r^{2}(H_{xz}^{2}+H_{y}^{2})}\\ +\left[{\left(\frac{1-\frac{2\chi^{\prime }}{3}{\left(\frac{r_{p}}{r}\right)}^{3}}{1+\frac{\chi^{\prime }}{3}{\left(\frac{r_{p}}{r}\right)}^{3}}\right)}^{2}-1\right]\frac{\left[(r^{2}+r_{y}^{2})H_{xz}^{2}+2(r^{2}-r_{y}^{2})H_{y}^{2}\right]}{2r^{2}(H_{xz}^{2}+H_{y}^{2})}\} \end{array}$$ (8)

where $m=4\pi r_{p}^{3}\chi^{\prime }\mu_{s}H/3$ is the induced magnetic dipole because the real part of the volume susceptibility χ′ is much larger than its imaginary part χ′′, *m*_0_ = ∣*m*∣, and *r* is the separation between particle *i* and *j*, *r* = ∣*r_ij_*∣ = (*r_x_*, *r_y_*, *r_z_*). The total energies can be calculated by summing the pair interactions over all particles *E* = ∑ ⟨*E_ij_*⟩. To obtain the coordinates of magnetic particles densely packed onto a spherical interface, we use 3ds Max GeoSphere with the icosahedron mesh method (*34*). In this, the distance between particles is chosen to be 1.1 μm to ensure no overlap between neighboring particles. Figure 4C shows the calculated energies per particle for the polar versus equatorial configurations at different surface coverage under the two different magnetic fields. Here, the energy is lower for the polar configuration under the 2D rotating field, but the equatorial configuration has lower energies upon the superposition of a DC field. These results are consistent with both experimental observations and the qualitative analysis based on pair interactions. Although the AC and DC field strengths are the same in Fig. 4A, it can be shown that the interaction (Eq. 8) energy between neighboring particle pairs is lower when accumulated at the equator than at the pole. Because the interaction due to the AC field is time averaged while the interaction due to the DC field is static, the magnitudes of those interactions are quantitatively different.

By changing the surface particle distribution, we can effectively tune the traction and speed of rolling droplets. In the fully covered case, droplets can translate at 30 to 100 μm/s; however, for sparsely covered droplets with a polar configuration, the propulsion speed can be as low as a few micrometers per second. Figure 4A shows that the same droplet with the equatorial configuration translates ~4× faster than the polar configuration (movie S4). As shown in Fig. 4D, at the same surface coverage, the traction enhancement, the ratio of γ between the equatorial and polar configurations, can be as high as 17. This enhancement can be attributed to two factors. First, the angular rotation speed ω_p_ of the equatorial configuration is smaller than the polar configuration for the same surface coverage. This is due to the interference of new, orthogonal torques generated by adding a DC component to the applied field, making the droplets wobble during rolling (movie S5). Thus, ω_p_ is smaller than that with a purely *x-z* rotating field, where the particles adopt the polar configuration. Even when polar and equatorial droplet configurations roll at the same ω_p_, the traction of the equatorial configuration is still much larger. During rolling, the solid magnetic particles accumulated at the equator are adjacent to the wall. Therefore, similar to the fully covered case, its friction force is substantially increased because of Hertzian deformation.

### pH-triggered cargo delivery

**Fig. 5.**
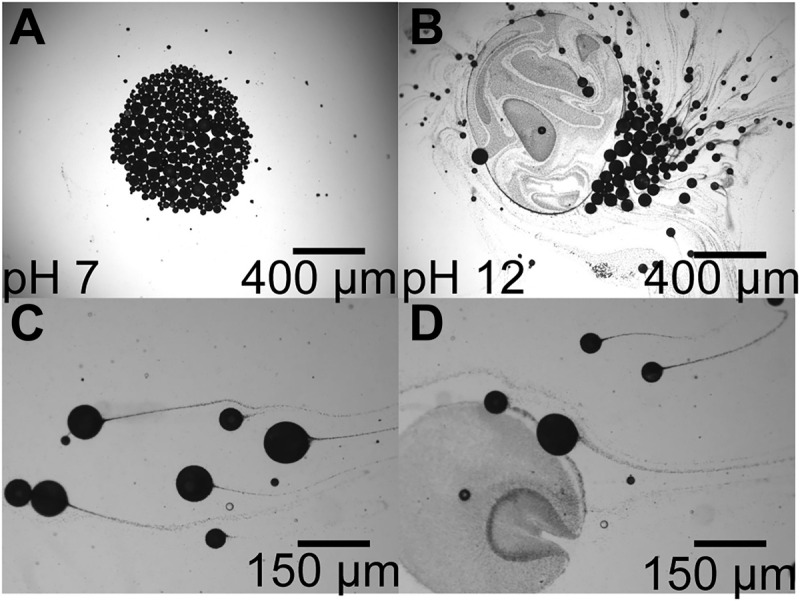
Destabilization of soft Pickering μbots. (**A**) Concentrated Pickering emulsion droplets at pH 7 and (**B**) at pH 12, where (**C**) particles leave the droplet interface forming long comet-like tails, eventually causing the droplets to (**D**) burst.

Soft μbots allow for the encapsulation of soluble cargo within the liquid core. Compared with rigid μbots, where cargo is typically attached to the μbot surface, the loading in soft μbots is potentially substantially higher. Because the liquid core can be either oil or water based, potentially reactive formulations used in targeted drug delivery could be encapsulated. With drug delivery as a potential application, we demonstrate that, depending on their surface energy, particles can be destabilized for the triggered release of the encapsulants. For example, with a sudden pH increase from 7 to 12, we show in Fig. 5 (A to D) that magnetic surface particles leave the o/w interface as long comet-like tails until the droplets burst (movie S6). Driving this process are the carboxyl functional groups coated on the particles. They are fully protonated in high pH environments, switching from amphiphilic to hydrophilic and moving into the water phase. As a result, the Laplace pressure can no longer sustain the increase of interfacial tension caused by the loss of surface particles, leading to droplet destabilization. Such a stimulus-triggered delivery mechanism is demonstrated in movie S7, where the Pickering emulsion droplets roll along at mild pH but destabilize when reaching high pH regions. Here, the droplets are confined within a capillary at pH 7 with tissue paper soaked with pH 13 water plugging one end. Once the droplets reach the end, particles gradually leave the interface destabilizing the bursting droplet.

We note that using pH to trigger the destabilization of μbots and release encapsulants can have potential biomedical applications. For example, it is widely accepted that the pH of cancer cells is slightly acidic compared with normal cells (6.4 to 7.0 versus 7.2 to 7.5), and studies have been performed to make polymeric nanoparticles responsive in this narrow pH range (*35*). In principle, one can tailor the surface functionalities of the magnetic particles to destabilize them within this pH range. In addition, other means, such as a high-frequency AC magnetic field (e.g., hyperthermia), can also be combined with thermoresponsive polymeric coatings [e.g., poly(*N*-isopropyl acrylamide)] on the magnetic particles and used as a potential strategy for destabilizing Pickering emulsions and releasing cargo in in vivo environments.

## DISCUSSION

We demonstrate the rolling of liquid Pickering emulsion droplets as soft μbots. Compared with their rigid counterparts, soft droplets have higher traction due to the greater friction force associated with their surface deformation and the larger Hertzian-like contact areas with adjacent wall surfaces. Moreover, we can use magnetic fields to reconfigure the particle distribution over the droplet surface when the droplets are partially covered. Under an AC field, particles accumulate at the poles while they redistribute toward the equator when a DC field is added orthogonally. As a result, the translation speed and traction can be tuned over a wide range depending on surface particle configurations. Last, we have shown that soft μbots can be triggered by pH change to destabilize and release cargo due to the departure of particles from the interface into the aqueous phase. Our work reveals the difference between deformable and rigid μbots in rolling, demonstrating the potential advantages of using soft μbots for cargo encapsulation, transport, and delivery.

With regard to other environments and in addition to flat surfaces, soft droplets can translate on curved walls (movie S8). In this, a droplet can roll 90° either clockwise or counterclockwise along a cylindrical capillary, demonstrating its ability to overcome gravity/buoyancy. Droplets can also roll along the longitudinal direction of the same capillary with curvature comparable to that of the droplet. We have also tested droplet rolling on textured surfaces using PDMS replicas of diffraction gratings (fig. S6 and movie S9). Tractions as high as 0.85, approaching the limit of dry friction with no slip, were measured here because of droplet compliance and Hertzian contact with the substrate leading to minimal liquid flux within the gap. These results demonstrate both the notable potential of soft μbots and how μbot and substrate properties can profoundly affect μbot rolling.

We note that the Young’s moduli of soft droplets are comparable to those of biological cells/tissues and orders of magnitude smaller than other types of soft μbots based on elastomeric composites (table S1); therefore, we expect no damage to tissues or blood vessels. These μbots are stable in standard phosphate-buffered saline solutions (pH 7.4 and high salt concentrations) but can also be destabilized when the pH changes to 12 (movie S10). We also note that the translation speed of soft droplet-based rollers is lower than the rigid μbots that we have previously studied (*22*). This is due to the availability of magnetic particle loading only at the droplet interface, resulting in an overall smaller magnetic torque and leading to a trade-off between droplet size, which dictates drug-loading capacity, and μbot velocity. In addition and although we have used pH change to trigger emulsion destabilization, alternative methods to destabilize surface particles, such as light- or magnetic field–induced hyperthermia (*36*), are also possible.

## MATERIALS AND METHODS

### Materials

Superparamagnetic Dynabeads MyOne carboxylic acid (2*a* = 1 μm, carboxylic acid–functionalized, particle concentration = 10 mg/ml, zeta potential = −60 mV; Thermo Fisher Scientific), 1-ethyl-3-(3-dimethyl aminopropyl) carbodiimide hydrochloride (EDC), *N*-hydroxysuccinimide (NHS), decane, Pluronic F-127, and decylamine were purchased from Sigma-Aldrich and used as received. Dimethyl sulfoxide (DMSO; ≥99.7%) and ethanol (200 proof) were purchased from Fisher Scientific and used as received. 184 Silicon Elastomer Kit was purchased from Dow Inc. (SYLGARD) and used as received. Oil-based ferrofluids EMG 900 was purchased from Ferrotec.

### Synthesis of amphiphilic particles and fabrication of Pickering emulsions

Before surface modification, 400 μl of Dynabeads solution from the stock solution was washed with DMSO to remove residual water and surfactants. To activate the carboxyl-functional groups, we mixed this solution with 50 ml of DMSO and reacted with EDC/NHS (3.8/2.3 mg/ml) reagents for 4 hours. We then added 0.24 μl of decylamine to modify the Dynabeads surfaces with alkyl chains for 24 hours. After surface modification, the Dynabeads were washed with DMSO thrice, ethanol twice, and water twice, after which the modified Dynabeads were dispersed in deionized water at a concentration of 1 mg/ml. Last, to make stable Pickering emulsions, we added 400 to 800 μl of decane to the 5 ml of aqueous Dynabeads (particle concentration, 1 wt %) solution and agitated the mixture for 2 min.

### Fabrication of rigid Pickering spheres and soft ferrofluid droplets

We first mixed 1 ml of PDMS precursor and curing agent (4:1 by weight) with 1 ml of hexane. The mixture was then added to a 5-ml modified Dynabeads aqueous solution (1 wt %). After vortex mixing for 2 min, stable Pickering emulsions with a PDMS liquid core and hexane were fabricated. After evaporating hexane in the resulting emulsions, we subsequently heated the emulsions at 70°C for 1 hour to cure the PDMS. This process yielded Dynabeads-coated and rigid PDMS spheres of approximately the same size as the Pickering droplets with a decane liquid core. Sucrose solutions with different concentrations were used to separate the rigid Dynabeads-coated Pickering spheres of different densities. The soft ferrofluid droplets were made by emulsifying 400 μl of ferrofluids with 10 ml of 1 wt % F-127 aqueous solution.

### Characterization

The bead zeta potential before and after surface modification was measured with a Zetasizer (Zeta PALS). The morphologies of rigid Pickering spheres with solid PDMS cores were characterized by scanning electron microscopy (JOEL 700FESEM). On an Olympus IX 70 optical microscope, the translational and rotational speeds of soft droplets and rigid spheres were measured on the basis of images captured by a black-and-white camera (EPIX Inc., SV643M) at 50 frames/s. The rotation of spheres was measured by tracking surface defects.

### Magnetic field setup

The magnetic field was generated by four orthogonal copper solenoid coils (fig. S3) with length of 51 mm, inner diameter of 50 mm, and 200 or 400 turns. All coils were installed on an aluminum crossing frame (*37*). One coil with 100 turns, length of 25 mm, and inner diameter of 50 mm was inserted between four orthogonal coils to apply a field along the *z* direction. Each coil was connected to an AC or DC power source, controlled by in-house software (MuControl 1.0.4 version) (*37*) and an output card (National Instruments, NI-92630). Current detection for each coil was performed through a data acquisition card (NI USB-6009) by measuring the voltage across a 1-ohm resistor (ARCOL, HS150 1 RF) connected in series. The magnetic field strength was measured by an AC/DC Gauss Meter (MF-30K). To generate the 2D rotating magnetic field, we passed a 20-Hz AC through each coil oriented along the *x* and *z* directions.

### Numerical simulation

The droplets that we consider in the numerical simulations are densely coated by particles. Therefore, we treat the droplet surface implicitly and only track the positions of the particles. We do not consider any detailed interfacial hydrodynamics, such as contact line dynamics and meniscus dragging, in our simulations, nor do we account for multiple fluid phases. We simply treat the medium fluid as a bulk with constant viscosity η and use the fast Stokesian dynamics method described in (*38*) to account for hydrodynamic interactions between particles. This approximation is reasonable because the particles that we consider are jammed on the droplet interface and do not move much relative to each other, rendering interfacial corrections to the hydrodynamic mobility higher order (*39*, *40*). Furthermore, the literature has shown that even with the full resolution of a two-phase Stokesian fluid, corrections to the bulk hydrodynamic mobility are typically less than 10% (*41*).

We express the force balance on the group of particles straddling the surface of the Pickering droplet of radius *R* as

$$F^{\mathrm{hydro}}+F^{\mathrm{bend}}+F^{\mathrm{tension}}+F^{\mathrm{mag}}+F^{\mathrm{grav}}=0$$ (9)

The magnetic forces **F**^mag^ are used to drive the droplets. The interfacial forces **F**^hydro^, **F**^bend^, and **F**^tension^ constrain the particles and capture the droplet softness. The buoyant force **F**^grav^ balances **F**^bend^ to control deformation against the bottom wall where, for convenience, the droplet is assumed to be heavier than the medium. The hydrodynamic force **F**^hydro^ = **M**^−1^**U** gives the particle velocities in the suspension through the mobility matrix **M**. In our simulations, **M** is calculated using lubrication corrections to accurately resolve both near- and far-field interactions between particle pairs and a bottom wall (*38*).

To calculate the interfacial forces **F**^hydro^and **F**^bend^ on the particles, we use their positions in space as the vertices of a triangulated surface mesh representing the droplet. This surface mesh is recalculated at every time step using the “ball pivoting” algorithm (*42*) to avoid substantial mesh distortion as the colloids are displaced. Ball pivoting requires an estimation of the normal vector at each vertex or colloid position in our case, so at time *t*, we use the mesh from time *t* − ∆*t* to estimate the vertex normals as an area-weighted average of the nearest one-ring face-valued normals with the mesh given explicitly for the first step.

The surface mesh allows us to calculate bending/tension forces that mimic the interfacial forces given the particle positions **X***_i_*, *i* = 1, …, *N*_p_. Specifically, we use the discrete bending force described by (*43*)

$$F_{i}^{\mathrm{bend}}(t)=\frac{-\partial }{\partial X_{i}}\sum_{i}^{N_{p}}\frac{\kappa_{\mathrm{bend}}}{2}{\left(\frac{\left\|C_{i}\right\|}{A_{i}(t)}\right)}^{2}A_{i}(t)$$ (10)

and a slightly modified version of the surface tension force

$$F_{i}^{\mathrm{tension}}(t)=\sigma (1.0-A_{i}(t)/A_{i}^{0})C_{i}$$ (11)

$$F_{i}^{\mathrm{tension}}[X_{i}](t)=\frac{-\partial }{\partial X_{i}}\sum_{i}^{N_{p}}\frac{\sigma }{2}{\left(\frac{A_{i}(t)-A_{i}^{0}}{A_{i}^{0}}\right)}^{2}A_{i}^{0}$$ (12)

In the above equations, *A_i_*(*t*) is the vertex area at node *i* and time *t*, $A_{i}^{0}$ is vertex area at node *i* and time *t* = 0, **C***_i_* is the mean curvature normal at node *i* and time *t*, κ_bend_ is the bending modulus of the droplet, which is treated as a control parameter, σ ∝ *k*_B_*TN*_p_/8π*R*^2^ is a penalty parameter used to enforce a constant surface area on the droplets as an effective surface tension, and *k*_B_*T* is the thermal energy at 25°C. σ is chosen to be large enough that the surface area changes by less than 1% over the length of a simulation and small enough that a reasonable time step size can be used.

Particles on the surface of the droplets are packed densely enough to be nearly jammed. To capture this effect, we include spring forces between neighboring particles with rest lengths determined by the particle spacing at *t* = 0 and stiffness κ*_s_* = 2*k*_B_*T*. These spring forces also act to regularize the droplet mesh in time and prevent unphysical shearing or large local area deformations. It has been shown that particles on a fluid interface tend not to rotate from an applied torque (*44*). We capture this effect by imposing a strong penalty torque on each particle of the form

$${\text{τ}}_{i}^{\mathrm{align}}=C\sigma r_{p}({\hat{n}}_{i}\times D_{i}^{3})$$ (13)

where $D_{i}^{3}$ is one component of the orthonormal triad representing the *i*th particle’s orientation [here, the third component is used for consistency with our past work on filaments (*45*)], ${\hat{n}}_{i}$ is the *i*th vertex normal of the mesh, *r*_p_ = 0.5 μm is the radius of a colloid, and *C* = 8 is the strength of the penalty.

Following prior work (*45*), we model the magnetic forces between particle *i* and *j* as

$$F_{ij}^{\mathrm{mag}}=\frac{F_{0}}{{\bar{r}}_{ij}^{4}}\{2(\bar{m}\cdot {\hat{r}}_{ij})\bar{m}-[5{\left(\bar{m}\cdot {\hat{r}}_{ij}\right)}^{2}-1]{\hat{r}}_{ij}\}$$ (14)

where $F_{0}=3\mu_{s}m_{0}^{2}/4\pi r_{p}^{4}$, ${\hat{r}}_{ij}=r_{ij}/r_{p}$, *m*_0_ = *V*_0_χ′*H*_0_, $V_{0}=4\pi r_{p}^{3}f/3$, *f* is the fraction of the particle that is paramagnetic, and $\bar{m}=m/m_{0}$ is the dimensionless magnetic moment of the particle. For the simulations presented in this work, we take *F*_0_ = 1.75 × 10^−12^*N*, based on our actual experimental conditions. The complex part of the magnetic susceptibility χ′′ generates a magnetic torque on particle *i* (*45*)

$${\text{τ}}_{i}^{\mathrm{mag}}=\tau_{0}\hat{y}=\frac{4\pi r_{p}^{3}}{3\mu_{s}}B_{0}^{2}\chi^{\prime \prime }\hat{y}$$ (15)

Given that the particles on the surface of the droplets are constrained to effectively not rotate, the torque applied to them through the magnetic field gets translated into an effective tangential force of the form

$$F_{i}^{\tau ,\mathrm{mag}}=\frac{\tau_{0}}{r_{p}}{\hat{n}}_{i}\times \hat{y}$$ (16)

All parameters used in the numerical simulations are listed in Table 1.

**Table 1.** Parameters and values used in the simulations.

Parameter	Value	Unit
Viscosity, η	10^−3^	Pa·s
Particle radius, *r*_p_	0.5	μm
Drop radius, *R*	4.6 to 19.8	μm
Buoyant force, *N*_p_*m*_e_*g*	83.5 to 331.5	pN
Magnetic force, *F*_0_	1.75	pN
Magnetic torque, τ_0_	0.435	aJ
Effective surface tension, σ	17.6	aJ/μm^2^
Bending modulus, κ_bend_	41.7 to 228.3	aJ

The error bars for markers along the solid black curve shown in Fig. 3B are calculated using traction values from droplets with the same $\bar{a}$ but different values of κ_bend_ and *m*_e_*g*. Specifically, the lower bound of the error bar corresponds to droplets with a 25% reduction in buoyant force and 25% increase in bending force, while the upper bound of the error bar corresponds to droplets with a 25% increase in buoyant force and 25% decrease in bending force. The low variance in the error bars demonstrates that droplet traction is primarily controlled by the dimensionless softness $\bar{a}$. Because of computational constraints, error bars for markers along the dashed line (those measuring the effect of increased drop radius on traction) are calculated conservatively by measuring the relative percentage difference between two droplets with $\bar{a}$ = 2.60, *R* = 11.66 μm, κ_bend_ = 59.73, 238.91 aJ, and *m*_e_*g* = 119.46, 477.82 pN and then using this percentage difference as the error for all markers along the dashed line. A 4× disparity in bending/buoyant forces was used here to ensure conservative error bars with a resulting relative traction difference = 2|γ_1_−γ_2_|/|γ_1_+γ_2_| = 3.7%.

## Supplementary Materials

### This PDF file includes:

Supplementary Text Figs. S1 to S6 Table S1 Legends for movies S1 to S10 References
