# Supplementary material for: Rolling of soft microbots with tunable traction
Source: Sci Adv. 2023 Apr 21;9(16):eadg0919. doi: 10.1126/sciadv.adg0919 (PMC10121164; doi:10.1126/sciadv.adg0919)
Supplement: Supplementary file 1 — Supplementary Text Figs. S1 to S6 Table S1 Legends for movies S1 to S10 References [file sciadv.adg0919_sm.pdf]

Science Advances
AAAS

Supplementary Materials for

Rolling of soft microbots with tunable traction

Yan Gao *et al.*

Corresponding author: David W. M. Marr, dmarr@mines.edu; Ning Wu, ningwu@mines.edu

Sci. Adv. **9**, eadg0919 (2023)
DOI: 10.1126/sciadv.adg0919

The PDF file includes:

Supplementary Text
Figs. S1 to S6
Table S1
Legends for movies S1 to S10
References

Other Supplementary Material for this manuscript includes the following:

Movies S1 to S10

Supplementary Text

Section S1. Estimation of the gap between a rigid Pickering sphere and the glass substrate

The gap between a rigid Pickering sphere and glass wall h was calculated with a balance between gravitational F_g and electrostatic forces F_e (46). The gravitational force F_g is

$$F_g = \frac{4}{3}\pi R^3(\rho_d - \rho_s)g \tag{S1}$$

where R is the sphere radius, g is the gravitational constant, ρ_d is the effective density of the Pickering droplet, and ρ_s is the solvent density. The electrostatic force is

$$F_e = 64\pi\varepsilon_s\varepsilon_0\kappa R\left(\frac{k_BT}{e}\right)^2 \tanh\left(\frac{e\psi_1}{4k_BT}\right)\tanh\left(\frac{e\psi_2}{4k_BT}\right)e^{-\kappa h} \tag{S2}$$

where κ^{-1} is the Debye length, h is the separation between the sphere and the glass substrate, ε_0 is the vacuum permittivity, ε_s is the dielectric constant of the solvent, k_B is Boltzmann's constant, and ψ_1 and ψ_2 are the surface potentials of the decylamine-modified beads and the glass slide, respectively (47). A plot of ln(*h/R*) vs. *R* is shown in Fig. S4, which reveals a scaling relationship of $-\ln(h/R) \propto R^{-0.36}$.

Section S2. Estimation of the soft Pickering droplet traction

Referring to Fig. 2D, we can write the momentum conservation equation along the *x*-axis as

$$-\frac{dP}{dx} + \eta\frac{\partial^2 v_x}{\partial z^2} = 0 \tag{S3}$$

with the following boundary conditions

$$z = 0,\ v_x = 0;\ \text{and}\ z = h_0,\ v_x = U - V_s \tag{S4}$$

where U is the translation velocity of the droplet and V_s is the tank-treading velocity of the beads at the surface $V_s = \omega_p R$. Assuming that the pressure depends on x only, integration of Eq. (S3) yields a velocity profile resulting from a combination of Couette and Poiseuille flows

$$v_x = (U - V_s)\frac{z}{h_0} + \frac{1}{2\eta}\frac{dP}{dx}h_0^2\left[\frac{z}{h_0} - \left(\frac{z}{h_0}\right)^2\right]. \quad \text{(S5)}$$

By continuity, the flux of liquid within the gap $q = \int_0^{h_0} v_x dz$ remains constant at steady state; therefore, the pressure is

$$P(x) = P_0 - \int_0^x \left[\frac{6\eta}{h_0^2}(U - V_s) - \frac{12\eta q}{h_0^3}\right] dx. \quad \text{(S6)}$$

The shear force on the contact area of the droplet is approximately

$$F_{x,shear} \approx 2a\int_0^{2a} \tau_{zx}|_{z=h_0} dx = 2a\int_0^{2a} \eta\frac{\partial v_x}{\partial z}\bigg|_{z=h_0} dx = 4a^2\left[\frac{4\eta(U - V_s)}{h_0} - \frac{6\eta q}{h_0^2}\right] \quad \text{(S7)}$$

while the pressure force is approximately

$$F_{x,p} \approx 4a^2[P(0) - P(2a)] = 4a^2\left[\frac{12\eta(U - V_s)a}{h_0^2} - \frac{24\eta qa}{h_0^3}\right]. \quad \text{(S8)}$$

Since the droplet is force-free, the balance of pressure and shear forces yields a simple relationship between the droplet's translation velocity *U* and the tank-treading velocity of beads on the surface V_s

$$U - V_s = \frac{q}{h_0}\frac{\left(\frac{24a}{h_0} + 6\right)}{\left(\frac{12a}{h_0} + 4\right)} \quad \text{(S9)}$$

In the limit of $a \gg h_0$, $U - V_s = U - \omega_p R = 2q/h_0$ and, when q is small, $U \sim \omega_p R$, the traction approaches one.

<u>Section S3. Calculation of the surface particle energies in different configurations</u>

The magnetic energy of surface particles with different configurations can be calculated via

$$E = \frac{1}{2}\sum_{i=1}^{N}\sum_{j=1}^{N}\langle E_{ij}\rangle \quad \text{(S10)}$$

where $\langle\cdot\rangle$ indicates time-averaging. In this, E_{ij} is the magnetic dipolar interaction between a pair of paramagnetic particles with mutual interaction described by (48)

$$E_{ij}(r,\theta) = \frac{m^2}{4\pi\mu_0 r^3\left[1-\frac{2\chi}{3}\left(\frac{R}{r}\right)^3\right]^2}\left\{1-3\cos^2\theta+\left[\left(\frac{1-\frac{2\chi}{3}\left(\frac{R}{r}\right)^3}{1+\frac{\chi}{3}\left(\frac{R}{r}\right)^3}\right)^2-1\right]\sin^2\theta\right\} \quad \text{(S11)}$$

where $\mathbf{m} = 4\pi r_0^3\chi\mu_0\mathbf{H}$ is the induced dipole moment on one particle and $m = |\mathbf{m}|$, $\mathbf{r}_{ij} = \mathbf{r}_j - \mathbf{r}_i$ is the position vector between particles i and j, $r = |\mathbf{r_{ij}}|$, and θ is the angle between the vector $\mathbf{r}_{ij}$ and the magnetic field $\mathbf{H}$, $cos\theta = \frac{\mathbf{r}\cdot\mathbf{H}}{|\mathbf{r}||\mathbf{H}|}$. For a 3D precessing magnetic field of the form $\mathbf{H} = H_{xz}\cos(\omega_M t)\hat{\mathbf{x}} + H_y\,\hat{\mathbf{y}} + H_{xz}\sin(\omega_M t)\hat{\mathbf{z}}$, the time-averaged pair interaction can be obtained by integrating and normalizing E_{ij} over one period $2\pi/\omega_M$.

$$\langle E_{ij}(r)\rangle = \frac{m_0^2}{4\pi\mu_0 r^3\left[1-\frac{2\chi}{3}\left(\frac{r_p}{r}\right)^3\right]^2} \cdot\left\{1-\frac{3\left[(r^2-r_y^2)H_{xz}^2+2r_y^2H_y^2\right]}{2r^2\left(H_{xz}^2+H_y^2\right)} + \left[\left(\frac{1-\frac{2\chi}{3}\left(\frac{r_p}{r}\right)^3}{1+\frac{\chi}{3}\left(\frac{r_p}{r}\right)^3}\right)^2-1\right]\frac{\left[(r^2+r_y^2)H_{xz}^2+2\left(r^2-r_y^2\right)H_y^2\right]}{2r^2\left(H_{xz}^2+H_y^2\right)}\right\} \quad \text{(S12)}$$

where r_y and r_z are the *y*- and *z*-component of $\boldsymbol{r}_{ij}$, respectively.

To place the particles at the droplet interface, we consider dense packing of surface particles either at the pole or the equator with the coordinates obtained by the icosahedron mesh method. Here, the sphere surface was separated into uniform isosceles triangles and a geosphere mesh was created with the software 3ds Max. The separation between neighboring particles was chosen as 1.1 μm with a local packing of 0.9. With identified particle coordinates for a specific

configuration, we calculated the total energy of the system by summing all pair interactions based on Eq. (S12).

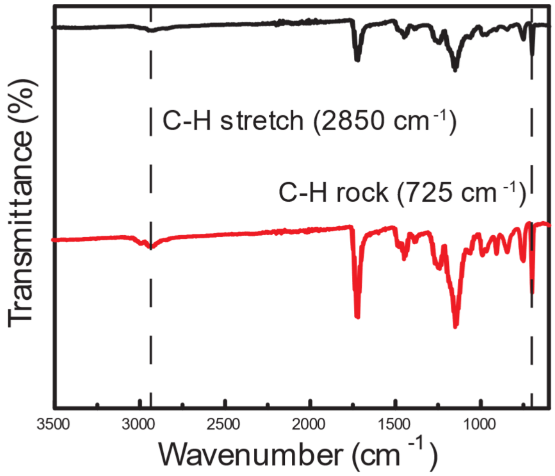

Figure S1. FTIR of magnetic particles before (black line) and after (red line) surface modification with decylamine.

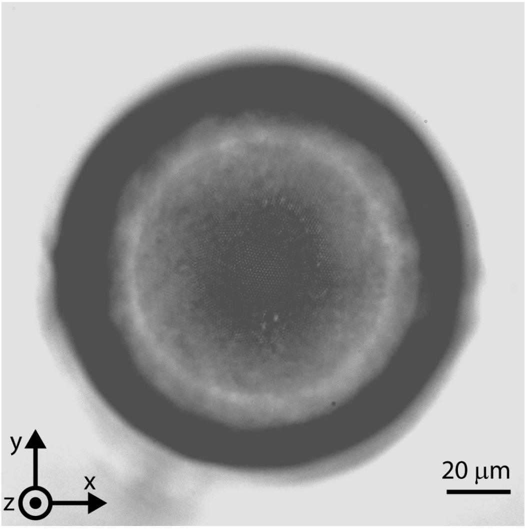

Figure S2. Microscopic image of the deformed droplet. A focused plane of close-packed particles can be seen clearly.

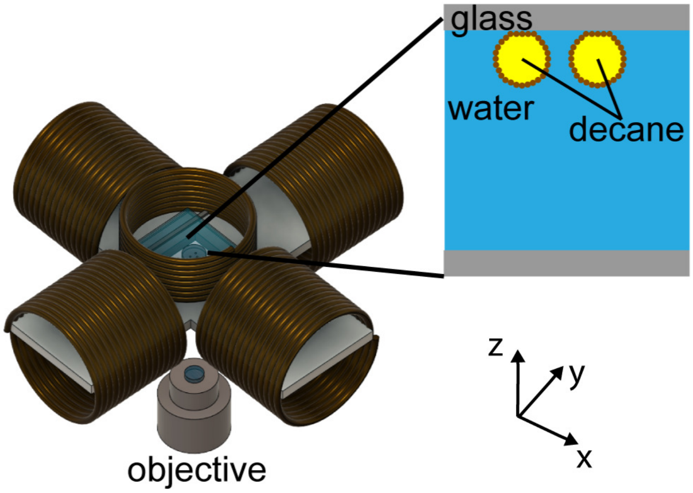

Figure S3. Experimental setup for studying the rolling of magnetic Pickering droplets. The applied field strength along each axis is 0.5 – 4.5 mT with a frequency f = 1–50 Hz (37).

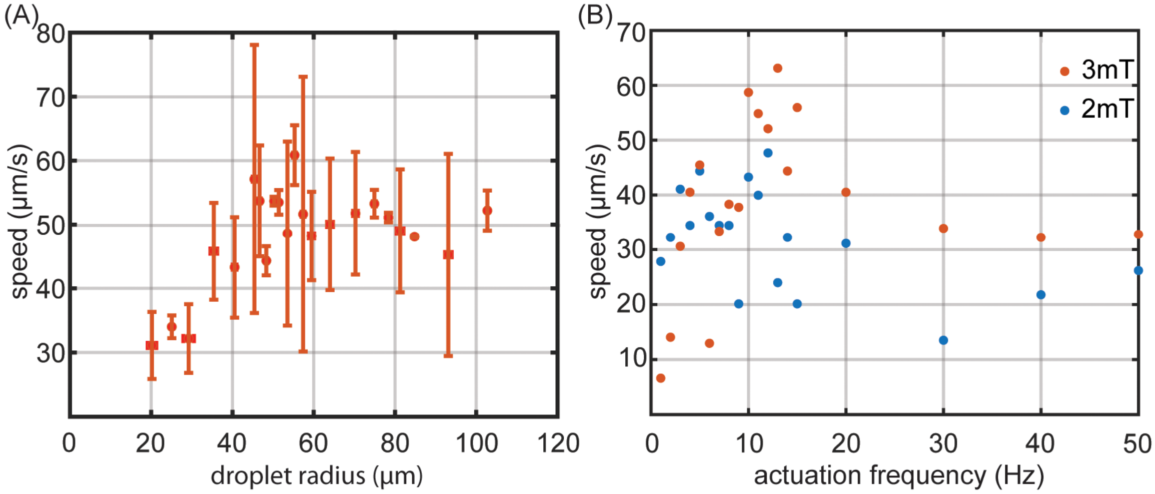

Figure S4. Translational speed of soft droplets with different radii and at different actuation frequencies. (A) The dependence of droplet translational speed on radius. (B) The speed of a 17.5 μm (radius) droplet under different actuation frequencies and magnetic field strengths.

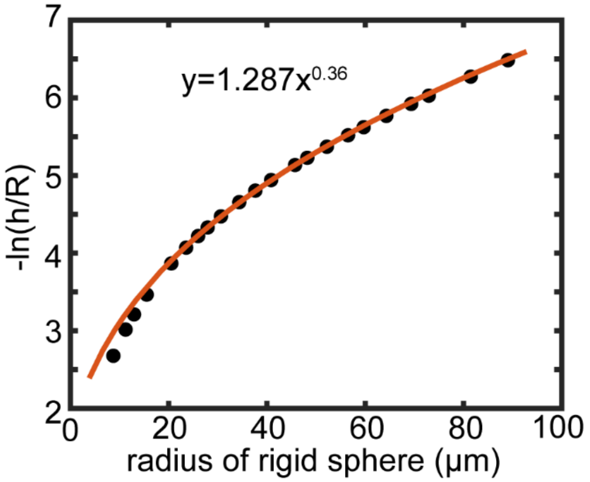

Figure S5. The dependence of -ln(*h/R*) on the radius of a rigid sphere. The solid spheres are calculated results and the red line is fitted with a power function.

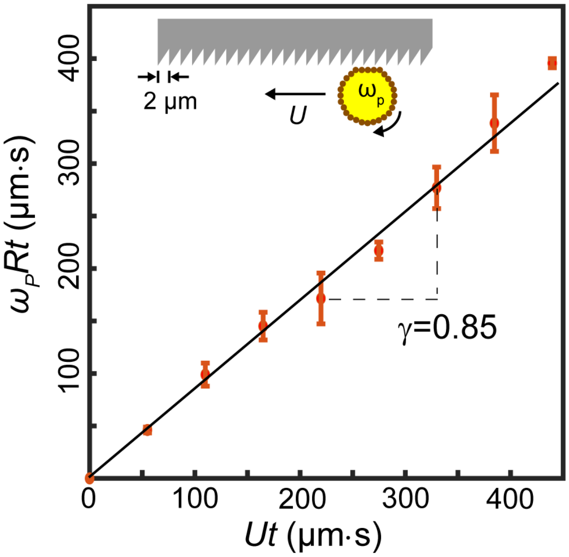

Figure S6. Soft Pickering droplet rolling near a textured surface with traction ~ 0.85 under a 2.5 mT rotating field. Inset: Schematic of the textured surface (PDMS replica of a diffraction grating) with 2 μm pitch and 4 μm peak-valley depth. R is the droplet radius, U_t is the translational velocity, and t is time.

Table S1. Comparison of Young's moduli between different soft μbots and biological cells/tissues.

Cell/soft μbots	Young's modulus (kPa)	Reference
Endothelial cells	0.218 ± 0.038	(49)
MCF-7 cells	2.4 ± 0.2	(50)
Mouse fibroblast Balb/c3T3 clone A31-1-1 cells	0.5-1.6	(51)
Hydrogel bilayer microgrippers, Polymer-coated Au wire motor	1.6×10^3-1.6×10^5	(52)(53)
PNIPAM or PDMS doping with MNPs, PNIPAM Janus motor, Polymeric spermbots	6×10^3-10^6	(54)(55) (56)(57)
Pickering emulsion droplets	0.139	This work

Movie S1. A fully covered soft Pickering emulsion droplet rolling near a glass substrate (field strength = 1.5 mT with video slowed down 3×). We focus on the contact area of the liquid sphere with the glass substrate, where the hexagonal packing of 1 μm beads indicates that the deformation area remains constant as the droplet rolls.

Movie S2. The rolling of soft and rigid Pickering droplets under a 2D rotating field (field strength =2.5 mT). Because the soft droplet is lighter, it floats to the top, while the heavier rigid droplet sinks to the bottom. As a result, they roll in opposite directions under the same rotating magnetic field.

Movie S3. Numerical simulation of rolling of a soft (yellow) and a rigid (blue) droplet under a 2D rotating magnetic field (field strength = 2.5 mT). The droplets settle down for the first 0.25 s, and then the magnetic field is applied. The movie is slowed down by 10×.

Movie S4. Rolling of 40% covered droplets with the equatorial (left) and polar configurations (right). The strengths of the AC and DC fields are 2.5 mT. Under the precessing field (DC+AC), surface particles accumulate at the equator and the droplets move faster and have higher traction.

Movie S5. Rolling and wobbling of partially covered soft droplets. The AC field strength is 1.5 mT at 5 Hz and the DC field strength is 1.5 mT. Wobbling is most apparent under 5 Hz.

Movie S6. Pickering emulsion droplets are stable in pH 7 environments. However, after tuning the pH to 12, surface beads become hydrophilic and leave the droplet interface inducing droplet rupture.

Movie S7. Fully covered droplets roll from the bottom to the top under a 2.5 mT AC magnetic field. The bottom region is pH 7, and the top is pH 12. Droplets roll smoothly in mild pH conditions. When the droplet reaches the high pH region, surface particles gradually leave the water-decane interface and the droplet bursts.

Movie S8. Droplet rolling on curved surfaces. A 66 μm (diameter) droplet rolling clockwise and counterclockwise 90° in a 200 μm inner diameter cylindrical capillary under a 3.0 mT *y-z* rotating magnetic field. A 100 μm droplet translating in the same capillary under a 3.0 mT *x-z* rotating magnetic field.

Movie S9. Droplet rolling near a textured surface (PDMS replica of an optical grating with 2 μm pitch and 4 μm peak-valley depth). The field strength is 2.5 mT, and the frequency is 20 Hz. Because the droplet is lighter than medium, it floats to the top and contacts with the textured surface.

Movie S10. Pickering emulsion droplets retain 95% stability in 1× PBS buffer solution. However, droplets become unstable and burst after adding a base solution to reach pH 12.

REFERENCES AND NOTES

1. P. Erkoc, I. C. Yasa, H. Ceylan, O. Yasa, Y. Alapan and M. Sitti, Mobile microrobots for active therapeutic delivery. *Adv. Ther.* **2**, 1800064 (2019).

2. J. Wang, W. Gao, Nano/microscale motors: Biomedical opportunities and challenges. *ACS Nano* **6**, 5745–5751 (2012).

3. D. Li, Y. Liu, Y. Yang, Y. Shen, A fast and powerful swimming microrobot with a serrated tail enhanced propulsion interface. *Nanoscale* **10**, 19673–19677 (2018).

4. H. Ceylan, J. Giltinan, K. Kozielski, M. Sitti, Mobile microrobots for bioengineering applications. *Lab Chip* **17**, 1705–1724 (2017).

5. X. Yang, N. Wu, Change the collective behaviors of colloidal motors by tuning electrohydrodynamic flow at the subparticle level. *Langmuir* **34**, 952–960 (2018).

6. F. Ma, S. Wang, D. T. Wu, N. Wu, Electric-field–induced assembly and propulsion of chiral colloidal clusters. *Proc. Natl. Acad. Sci. U.S.A.* **112**, 6307–6312 (2015).

7. H. Zeng, P. Wasylczyk, D. S. Wiersma, A. Priimagi, Light robots: Bridging the gap between microrobotics and photomechanics in soft materials. *Adv. Mater.* **30**, 1703554 (2018).

8. F. Cheng, R. Yin, Y. Zhang, C.-C. Yen, Y. Yu, Fully plastic microrobots which manipulate objects using only visible light. *Soft Matter* **6**, 3447–3449 (2010).

9. A. Aghakhani, O. Yasa, P. Wrede, M. Sitti, Acoustically powered surface-slipping mobile microrobots. *Proc. Natl. Acad. Sci. U.S.A.* **117**, 3469–3477 (2020).

10. X. Lu, K. Zhao, W. Liu, D. Yang, H. Shen, H. Peng, X. Guo, J. Li, J. Wang, A human microrobot interface based on acoustic manipulation. *ACS Nano* **13**, 11443–11452 (2019).

11. Y. Alapan, U. Bozuyuk, P. Erkoc, A. C. Karacakol, M. Sitti, Multifunctional surface microrollers for targeted cargo delivery in physiological blood flow. *Sci. Robot.* **5**, eaba5726 (2020).

12. U. Bozuyuk, Y. Alapan, A. Aghakhani, M. Yunusa, M. Sitti, Shape anisotropy-governed locomotion of surface microrollers on vessel-like microtopographies against physiological flows. *Proc. Natl. Acad. Sci. U.S.A.* **118**, e2022090118 (2021).

13. K. J. Widder, A. E. Senyei, D. F. Ranney, In vitro release of biologically active adriamycin by magnetically responsive albumin microspheres. *Cancer Res.* **40**, 3512–3517 (1980).

14. K. Leong, J. Kost, E. Mathiowitz, R. Langer, Polyanhydrides for controlled release of bioactive agents. *Biomaterials* **7**, 364–371 (1986).

15. D. Jin, L. Zhang, Collective behaviors of magnetic active matter: Recent progress toward reconfigurable, adaptive, and multifunctional swarming micro/nanorobots. *Acc. Chem. Res.* **55**, 98–109 (2022).

16. F. Qiu, S. Fujita, R. Mhanna, L. Zhang, B. R. Simona, B. J. Nelson, Magnetic helical microswimmers functionalized with lipoplexes for targeted gene delivery. *Adv. Funct. Mater.* **25**, 1666–1671 (2015).

17. C. E. Sing, L. Schmid, M. F. Schneider, T. Franke, A. Alexander-Katz, Controlled surface-induced flows from the motion of self-assembled colloidal walkers. *Proc. Natl. Acad. Sci. U.S.A.* **107**, 535–540 (2010).

18. R. Dreyfus, J. Baudry, M. L. Roper, M. Fermigier, H. A. Stone, J. Bibette, Microscopic artificial swimmers. *Nature* **437**, 862–865 (2005).

19. J. Cui, T.-Y. Huang, Z. Luo, P. Testa, H. Gu, X.-Z. Chen, B. J. Nelson, L. J. Heyderman, Nanomagnetic encoding of shape-morphing micromachines. *Nature* **575**, 164–168 (2019).

20. H. Xie, M. Sun, X. Fan, Z. Lin, W. Chen, L. Wang, L. Dong, Q. He, Reconfigurable magnetic microrobot swarm: Multimode transformation, locomotion, and manipulation *Sci. Robot.* **4**, eaav8006 (2019).

21. T. O. Tasci, D. Disharoon, R. M. Schoeman, K. Rana, P. S. Herson, D. W. Marr, K. B. Neevas, Enhanced fibrinolysis with magnetically powered colloidal microwheels. *Small* **13**, 10.1002/smll.201700954 (2017).

22. T. Tasci, P. Herson, K. B. Neeves, D. W. M. Marr, Surface-enabled propulsion and control of colloidal microwheels. *Nat. Commun.* **7**, 10225 (2016).

23. A. J. Goldman, R. G. Cox, H. Brenner, Slow viscous motion of a sphere parallel to a plane wall—I motion through a quiescent fluid. *Chem. Eng. Sci.* **22**, 637–651 (1967).

24. D. Ahmed, C. Dillinger, A. Hong, B. J. Nelson, Artificial acousto-magnetic soft microswimmers. *Adv. Mater. Technol.* **2**, 1700050 (2017).

25. X. Chen, M. Fan, H. Tan, B. Ren, G. Yuan, Y. Jia, J. Li, D. Xiong, X. Xing, X. Niu, X. Hu, Magnetic and self-healing chitosan-alginate hydrogel encapsulated gelatin microspheres via covalent crosslinking for drug delivery. *Mater. Sci. Eng. C* **101**, 619–629 (2019).

26. E. Dintwa, E. Tijskens, H. Ramon, On the accuracy of the Hertz model to describe the normal contact of soft elastic spheres. *Granular Matter.* **10**, 209–221 (2008).

27. T. Supakar, A. Kumar, J. Marston, Impact dynamics of particle-coated droplets. *Phys. Rev. E* **95**, 013106 (2017).

28. B. Wu, C. Yang, Q. Xin, L. Kong, M. Eggersdorfer, J. Ruan, P. Zhao, J. Shan, K. Liu, D. Chen, D. A. Weitz, X. Gao, Attractive pickering emulsion gels. *Adv. Mater.* **33**, 2102362 (2021).

29. S. Krijt, C. Dominik, A. G. G. M. Tielens, Rolling friction of adhesive microspheres. *J. Phys. D Appl. Phys.* **47**, 175302 (2014).

30. T. Yang, T. O. Tasci, A. Tomaka, N. Wu, D. W. M. Marr, Microwheels on microroads: Enhanced translation on topographic surfaces *Sci.Robot.* **4,** eaaw9525 (2019).

31. Z. Wang, “Polydimethylsiloxane mechanical properties measured by macroscopic compression and nanoindentation techniques,” thesis, University of South Florida, St. Petersburg, FL (2011), p 78.

32. M. H. Essink, A. Pandey, S. Karpitschka, C. H. Venner, J. H. Snoeijer, Regimes of soft lubrication. *J. Fluid Mech.* **915**, A49 (2021).

33. V. A. Dahl, A. B. Dahl, R. Larsen, in *2014 2nd International Conference on 3D Vision* (IEEE, 2014), vol. 2, pp. 82–89.

34. M. T. Hart, The projection point geodesic grid algorithm for meshing the sphere. *J. Comput. Phys.* **454**, 110993 (2022).

35. N. Deirram, C. Zhang, S. S. Kermaniyan, A. P. Johnston, G. K. Such, pH-responsive polymer nanoparticles for drug delivery. *Macromol. Rapid Commun.* **40**, e1800917 (2019).

36. S. Sharma, N. Shrivastava, F. Rossi, N. T. K. Thanh, Nanoparticles-based magnetic and photo induced hyperthermia for cancer treatment. *Nano Today* **29**, 100795 (2019).

37. E. Roth, C. Zimmermann, D. Disharoon, T. Tasci, D. Marr, K. B. Neeves, An experimental design for the control and assembly of magnetic microwheels. *Rev. Sci. Instrum.* **91**, 093701 (2020).

38. B. Sprinkle, E. B. Van Der Wee, Y. Luo, M. M. Driscoll, A. Donev, Driven dynamics in dense suspensions of microrollers. *Soft Matter* **16**, 7982–8001 (2020).

39. A. Dani, G. Keiser, M. Yeganeh, C. Maldarelli, Hydrodynamics of particles at an oil–water interface. *Langmuir* **31**, 13290–13302 (2015).

40. A. Dani, M. Yeganeh, C. Maldarelli, Hydrodynamic interactions between charged and uncharged brownian colloids at a fluid-fluid interface. *J. Colloid Interface Sci.* **628**, 931–945 (2022).

41. J.-C. Loudet, M. Qiu, J. Hemauer, J. J. Feng, Drag force on a particle straddling a fluid interface: Influence of interfacial deformations. *Eur. Phys. J. E Soft Matter.* **43**, 13 (2020).

42. F. Bernardini, J. Mittleman, H. Rushmeier, C. Silva, G. Taubin, The ball-pivoting algorithm for surface reconstruction. *IEEE Trans. Vis. Comput. Graph.* **5**, 349–359 (1999).

43. C.-H. Wu, T. G. Fai, P. J. Atzberger, C. S. Peskin, Simulation of osmotic swelling by the stochastic immersed boundary method. *SIAM J. Sci. Comput.* **37**, B660-B688 (2015).

44. W. Fei, P. M. Tzelios, K. J. Bishop, Magneto-capillary particle dynamics at curved interfaces: Time-varying fields and drop mixing. *Langmuir* 10.1021/acs.langmuir.9b03119 (2020).

45. T. Yang, B. Sprinkle, Y. Guo, J. Qian, D. Hua, A. Donev, D.WM.Marr, N. Wu, Reconfigurable microbots folded from simple colloidal chains. *Proc. Natl. Acad. Sci. U.S.A.* **117**, 18186–18193 (2020).

46. J. N. Israelachvili, *Intermolecular and Surface Forces* (Academic Press, 2011).

47. W. B. Russel, W. Russel, D. A. Saville, W. R. Schowalter, *Colloidal Dispersions* (Cambridge University Press, 1991).

48. D. Du, "Novel dynamics and structures using paramagnetic colloids with rotating magnetic fields," thesis, Rice University, Houston, TX (2015).

49. S. Jalali, M. Tafazzoli-Shadpour, N. Haghighipour, R. Omidvar, F. Safshekan, Regulation of endothelial cell adherence and elastic modulus by substrate stiffness. *Cell Commun. Adhes.* **22**, 79–89 (2015).

50. S. Moreno-Flores, R. Benitez, M. dM Vivanco, J. L. Toca-Herrera, Stress relaxation and creep on living cells with the atomic force microscope: A means to calculate elastic moduli and viscosities of cell components. *Nanotechnology* **21**, 445101 (2010).

51. L. Sirghi, J. Ponti, F. Broggi, F. Rossi, Probing elasticity and adhesion of live cells by atomic force microscopy indentation. *Eur. Biophys. J.* **37**, 935–945 (2008).

52. S. Fusco, M. S. Sakar, S. Kennedy, C. Peters, R. Bottani, F. Starsich, A. Mao, G. A. Sotiriou, S. Pané, S. E. Pratsinis, D. Mooney, B. J. Nelson, An integrated microrobotic platform for on-demand, targeted therapeutic interventions. *Adv. Mater.* **26**, 952–957 (2014).

53. V. Garcia-Gradilla, S. Sattayasamitsathit, F. Soto, F. Kuralay, C. Yardımcı, D. Wiitala, M. Galarnyk, J. Wang, Ultrasound-propelled nanoporous gold wire for efficient drug loading and release. *Small* **10**, 4154–4159 (2014).

54. S. N. Tabatabaei, J. Lapointe, S. Martel, Shrinkable hydrogel-based magnetic microrobots for interventions in the vascular network. *Adv. Robot.* **25**, 1049–1067 (2011).

55. J. Liu S. Yu, B. Xu, Z. Tian, H. Zhang, K. Liu, X. Shi, Z. Zhao, C. Liu, X. Lin, G. Huang, A. A. Solovev, J. Cui, T. Li, Y. Mei, Magnetically propelled soft microrobot navigating through constricted microchannels *Appl. Mater. Today* **25**, 101237 (2021).

56. F. Mou, C. Chen, Q. Zhong, Y. Yin, H. Ma, J. Guan, Autonomous motion and temperature-controlled drug delivery of Mg/Pt-poly (N-isopropylacrylamide) Janus micromotors driven by simulated body fluid and blood plasma. *ACS Appl. Mater. Interfaces* **6**, 9897–9903 (2014).

57. V. Magdanz, M. Guix, F. Hebenstreit, O. G. Schmidt, Dynamic polymeric microtubes for the remote-controlled capture, guidance, and release of sperm cells. *Adv. Mater.* **28**, 4084–4089 (2016).
